# ﻿Two new cave-dwelling *Spiricoelotes* species (Araneae, Agelenidae) from Hubei, China

**DOI:** 10.3897/zookeys.1245.145389

**Published:** 2025-07-16

**Authors:** Hailun Chen, Jie Liu, Mian Wei

**Affiliations:** 1 Hubeiate Key Laboratory of Regional Development and Environmental Response, Faculty of Resources and Environmental Science, Hubei University, Wuhan 430062, China; 2 The State Key Laboratory of Biocatalysis and Enzyme Engineering of China, School of Life Sciences, Hubei University, Wuhan 430062, Hubei, China; 3 School of Nuclear Technology and Chemistry and Biology, Hubei University of Science and Technology, Xianning 437100, Hubei, China; 4 College of Life Sciences, Sichuan University, Chengdu, Sichuan 610064, China

**Keywords:** Biodiversity, cave, Coelotinae, COI, morphology, taxonomy

## Abstract

Two new cave-dwelling *Spiricoelotes* (Wang, 2002) species are described: *S.metyr***sp. nov.** (♂♀) and *S.zhengi***sp. nov.** (♂♀). Moreover, *Spiricoeloteszonatus* (Peng & Wang, 1997) is redescribed due to the high similarity to *S.zhengi***sp. nov.** All these three species are illustrated, and their collection localities are mapped. To ensure the correct gender match, the COI sequences data are provided.

## ﻿Introduction

The genus *Spiricoelotes* was established by Wang (2002), with the transfer of *Coeloteszonatus* Peng & Wang, 1997, the type species, to *Spiricoelotes*. It can be distinguished from other coelotine genera by the long, strongly convoluted spermathecae and the absence of epigynal teeth in females, and the strongly curved patellar apophysis, the elongated cymbial furrow, the absence of the dorsal apophysis of the conductor, and the slender, anteriorly extended, spiral conductor in males (Wang 2002). Currently, nine *Spiricoelotes* species have been described: *S.anshiensis* Chen & Li, 2016, *S.chufengensis* Chen & Li, 2016, *S.nansheensis* Chen & Li, 2016, *S.pseudozonatus* Zhang, Wang & Zhu, 2017, *S.taipingensis* Chen & Li, 2016, *S.xianheensis* Chen & Li, 2016, *S.xiongxinensis* Chen & Li, 2016, *S.urumensis* (Shimojana, 1989), and *S.zonatus* (Peng & Wang, 1997). Only *S.urumensis* was found in Japan, while the other eight *Spiricoelotes* species were found in China (World spider catalog 2025). Among these, six were discovered in caves (Chen et al. 2016); however, their large eyes and dark body coloration suggest that they are not truly troglobitic spiders.

During the examination of the coelotine specimens collected from the caves in Hubei Province, central China, two new *Spiricoelotes* species were discovered: *S.metyr* sp. nov. and *S.zhengi* sp. nov. The former inhabits the dark zones of caves and is characterized by reduced eyes and a pale body, which indicate its high adaptation to cave environments. In contrast, the latter resides at cave entrances and exhibits normal morphological traits and closely resembles *S.zonatus*. In this study, we describe these two new species and redescribe *S.zonatus*, with the detailed descriptions, color illustrations, and distribution maps of the new species. COI sequences were provided for verifying their gender match.

## ﻿Materials and methods

### ﻿Specimen sampling

Specimens studied here were collected from Hubei province, China, on 31 May 2023. All specimens were captured by hand and stored in 95% ethanol at −20 °C.

### ﻿Molecular data

To test the gender match of the two new speceis, two individuals of each species were selected from the examined materials for molecular data collection. Their first and second legs on the right were used to extract genomic DNA and sequence the gene fragments COI. The rest of the bodies were kept as vouchers. All molecular data were obtained from specimens collected at the type localities. Whole genomic DNA was extracted from tissue samples with the Universal Genomic DNA Kit (CWBIO, Beijing, China) following the manufacturer’s protocol for animal tissue. The COI gene fragments were amplified in 50 µL reactions. Primer pairs and PCR protocols are given in Table 1. New sequences from this study were deposited in GenBank (Table 2).

**Table 1.** The loci, primer pairs, and PCR protocols used in this study.

Locus	Annealing temperature/time	Direction	Primer	Sequence 5'→3'	Reference
COI	49 °C/15 s	F	LCO1409	GGTCAACAAATCATAAAGATATTGG	Folmer et al. 1994
		R	HCO2198	TAAACTTCAGGGTGACCAAAAAATCA	

**Table 2.** GenBank accession numbers for new DNA sequence data from two *Spiricoelotes* species.

Species	Sex/Identity	Stage	GenBank accession number	Sequence length
*S.metyr* sp. nov.	♂/LJ202369	Adult	PV670693	596
	♀/LJ202373	Adult	PV670692	660
*S.zhengi* sp. nov.	♂/LJ202385	Adult	PV670695	546
	♀/LJ202388	Adult	PV670694	546

### ﻿Morphological data

All specimens were examined using an Olympus SZX7 stereomicroscope. Male palps and female genitalia were dissected from the spider bodies for examination and photography. Epigynes were cleared with Proteinase K to reveal their internal structures. Photographs were captured with a Canon EOS 90D wide-zoom digital camera (8.5 megapixels) mounted on an Olympus BX43 compound microscope, and the images were stacked and processed using Helicon Focus v. 7.0.2 image-stacking software. Illustrations were made of the left palps. Leg measurements are provided as total length, including segments (femur, patella, tibia, metatarsus, tarsus). Measurements were taken exclusively from the left structures (e.g., pedipalps, legs).

Abbreviations are as follows:

**Morphological characteristics**

**ALE** anterior lateral eye

**AME** anterior median eye

**AME–ALE** distance between AME and ALE

**AME–AME** distance between AME and AME

**ALE–PLE** distance between ALE and PLE

**AME–PME** distance between AME and PME

**PLE** posterior lateral eye

**PME** posterior median eye

**PME–PLE** distance between PME and PLE

**PME–PME** distance between PME and PME

**Depositories of the specimens**

**CBEE**Centre for Behavioural Ecology and Evolution, College of Life Sciences, Hubei University;

**HNNU**College of Life Sciences, Hunan Normal University, Changsha.

## ﻿Taxonomy

### ﻿Family Agelenidae C.L. Koch, 1837

**Subfamily Coelotinae F.O. Pickard-Cambridge, 1893**

#### ﻿Genus Spiricoelotes

Taxon classificationAnimaliaAraneaeAgelenidae

Wang, 2002

76B15187-98CD-51F3-950C-46FE43E6955F

##### Type species.

*Spiricoeloteszonatus* (Peng & Wang, 1997).

##### Diagnosis.

With the inclusion of additional species in *Spiricoelotes*, the diagnostic characteristics of the genus are revised in this study. The males can be identified by the hook-shaped patellar apophysis, the slender, elongate conductor, the presence of a tegular ridge that is matched with the dorsal ridge (apophysis) of the conductor, and the reduction or absence of the median apophysis. The females can be identified by the absence of epigynal teeth, the reduction of the atrium, and deep hoods.

#### ﻿ Spiricoelotes metyr sp. nov.

Taxon classificationAnimaliaAraneaeAgelenidae

E00578BA-7DB4-5B90-A80E-B4440B5F45D7

https://zoobank.org/416A2FE3-BF89-414F-A4E6-2484EE1ED20F

**Figure 1.**
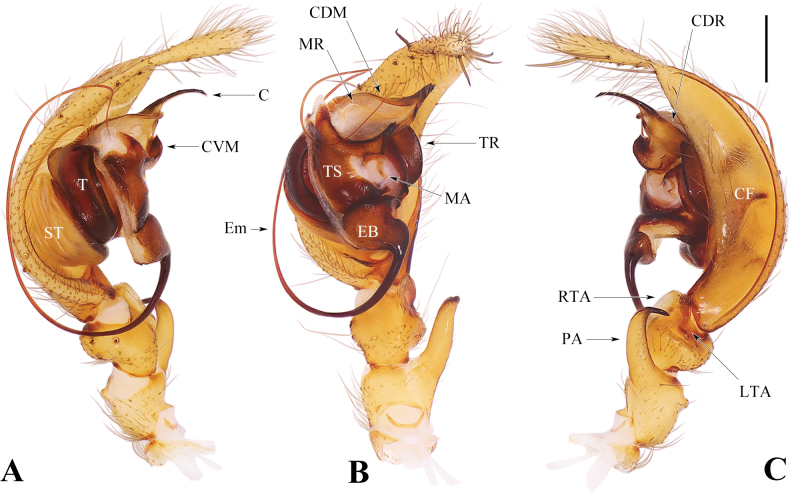
Male palp of *Spiricoelotesmetyr* sp. nov. **A**–**C.** Left palp: **A.** Prolateral view; **B.** Ventral view; **C.** Retrolateral view. Abbreviations: C = conductor; CF = cymbial furrow; CDM = dorsal margin of conductor; CDR = dorsal ridge of conductor; CVM = ventral margin of conductor; EB = embolic base; Em = embolus; LTA = lateral tibial apophysis; MA = median apophysis; MR = membranous ridge of dorsal margin of conductor; PA = patellar apophysis; RTA = retrolateral tibial apophysis; ST = subtegulum; T = tegulum; TR = tegular ridge; TS = tegular sclerite. Scale bar: 0.50 mm.

**Figure 2.**
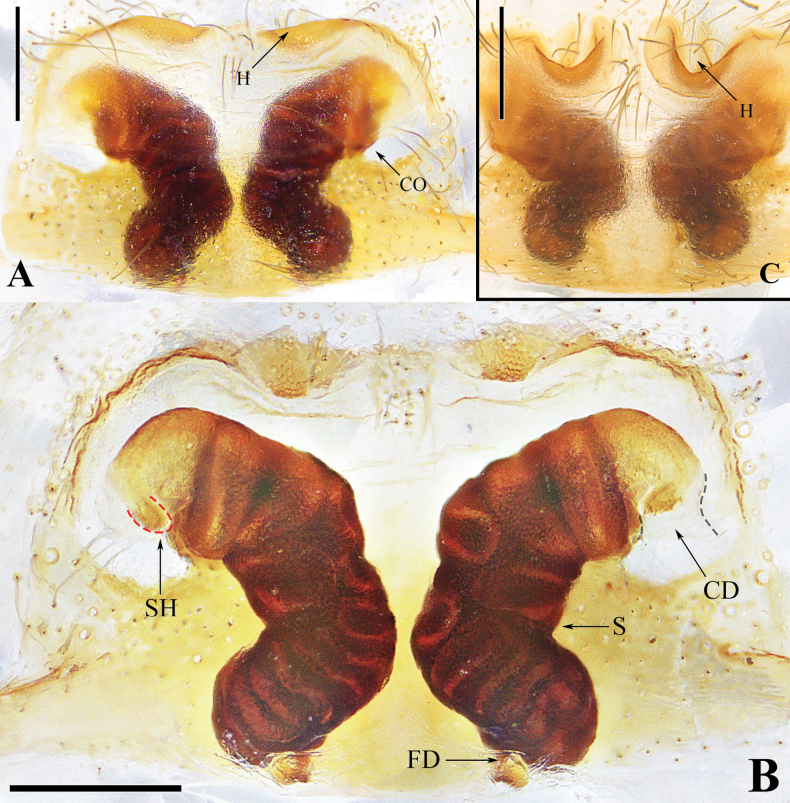
Epigynes of *Spiricoelotesmetyr* sp. nov. **A.** Epigyne, ventral view; **B.** Vulva, dorsal view; **C.** Variation of epigyne, ventral view. Abbreviations: CO = copulatory open; CD = copulatory duct; FD = fertilization duct; H = hood; S = spermatheca; SH = spermathecal head. Scale bars: 0.20 mm.

Figs 1 , 2 , 4A, B , 7A, B , 8

##### Type materials.

***Holotype*** • ♂ (CBEE, LJ202369), **China: *Hubei Province***: Xianning City, Chongyang County, dark zone of Daquan Cave, 29.5534°N, 114.2662°E, elevation: 124 m, 31.X.2023, Jian Chang, Mian Wei, Guoyuan Zhang and Haosiyi Zhu leg. ***Paratypes***: • 3♂♂3♀♀(CBEE, LJ202370–LJ202375), same data as holotype; • 4♂♂5♀♀(CBEE, LJ202376–LJ202384), **China: *Hubei Province***: Xianning City, Xianan District, dark zone of a nameless cave, 29.7715°N, 114.3122°E, elevation: 89 m, 10.XII.2023, Jian Chang, Guolong Huang and Mian Wei leg.

##### Etymology.

The species name is derived from “Metyr”, a character in the myth of Elden Ring, written by George R.R. Martin. Metyr is depicted as having a massive, finger-shaped body and living in an underground cave; this name refers to the shape of the spermathecae and the habitat of this new species. It is treated as a noun in genitive case.

##### Diagnosis.

**Figure 3.**
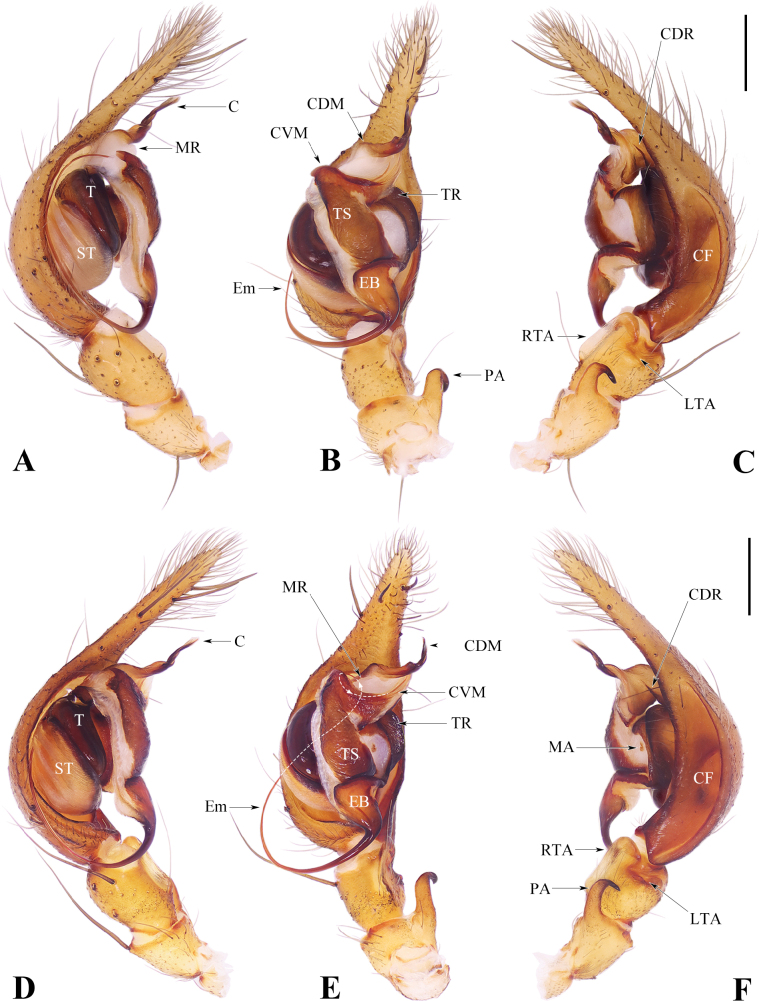
Male palps of *Spiricoeloteszhengi* sp. nov. and *Spiricoeloteszonatus*. **A–C.** Left palp of *Spiricoeloteszhengi* sp. nov. **D–F.** Left palp of *Spiricoeloteszonatus*. **A, D.** Prolateral view; **B, E.** Ventral view; **C, F.** Retrolateral view. Abbreviations: C = conductor; CF = cymbial furrow; CDM = dorsal margin of conductor; CDR = dorsal ridge of conductor; CVM = ventral margin of conductor; EB = embolic base; Em = embolus; LTA = lateral tibial apophysis; MA = Median apophysis; MR = membranous ridge of dorsal margin of conductor; PA = patellar apophysis; RTA = retrolateral tibial apophysis; ST = subtegulum; T = tegulum; TR = tegular ridge; TS = tegular sclerite. Scale bars: 0.50 mm.

**Figure 4.**
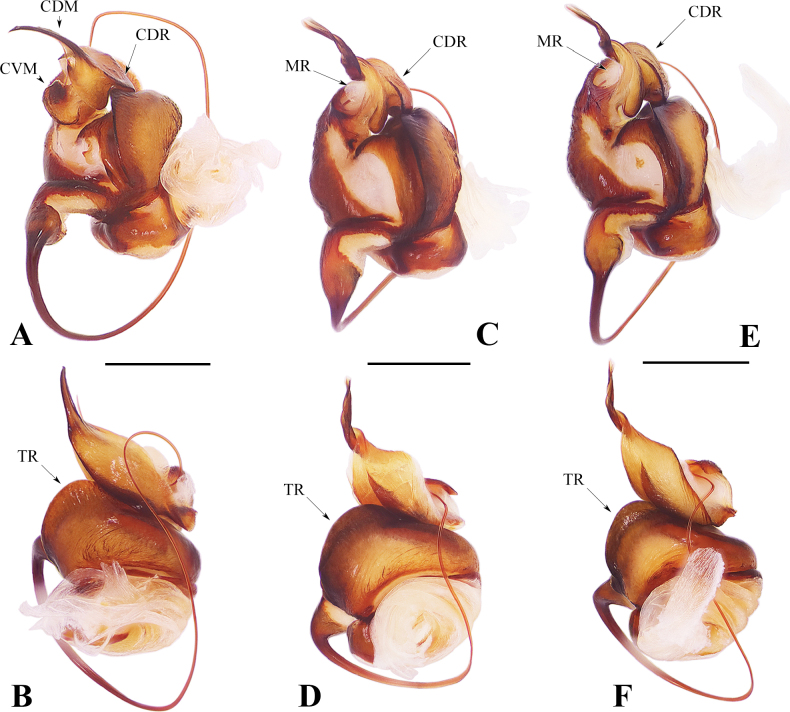
Male palpal bulbs of *Spiricoelotes* spp. **A, B.***S.metyr* sp. nov.; **C, D.***S.zhengi* sp. nov.; **E, F.***S.zonatus*. **A, C, E.** Retrolatral view; **B, D, F.** Dorsal view. Abbreviations: CDM = dorsal margin of conductor; CDR = dorsal ridge of conductor; CVM = ventral margin of conductor; MR = membranous ridge of dorsal margin of conductor; TR = tegular ridge. Scale bars: 0.50 mm.

**Figure 5.**
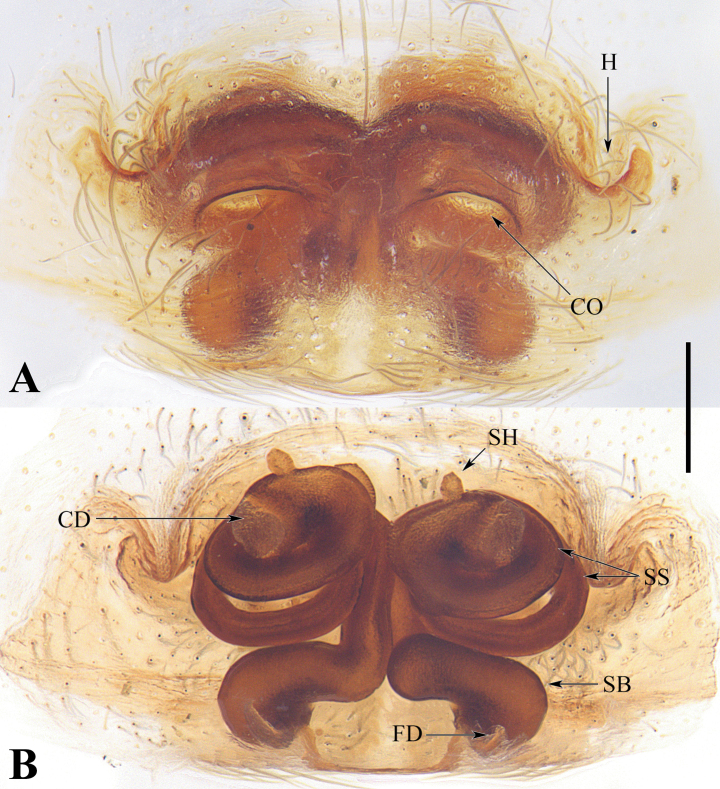
Epigyne of *Spiricoeloteszhengi* sp. nov. **A.** Epigyne, ventral view; **B.** Vulva, dorsal view. Abbreviations: CO = copulatory open; CD = copulatory duct; FD = fertilization duct; H = hood; SB = spermathecal base; SH = spermathecal head; SS = spermathecal stalk. Scale bar: 0.20 mm.

**Figure 6.**
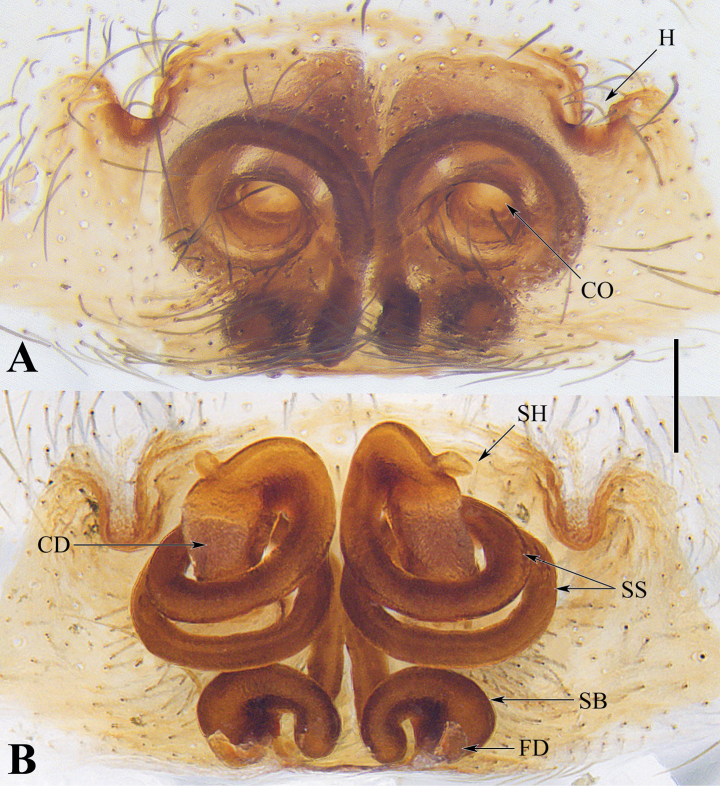
Epigyne of *Spiricoeloteszonatus*. **A.** Epigyne, ventral view; **B.** Vulva, dorsal view. Abbreviations: CO = copulatory open; CD = copulatory duct; FD = fertilization duct; H = hood; SB = spermathecal base; SH = spermathecal head; SS = spermathecal stalk. Scale bar: 0.20 mm.

**Figure 7.**
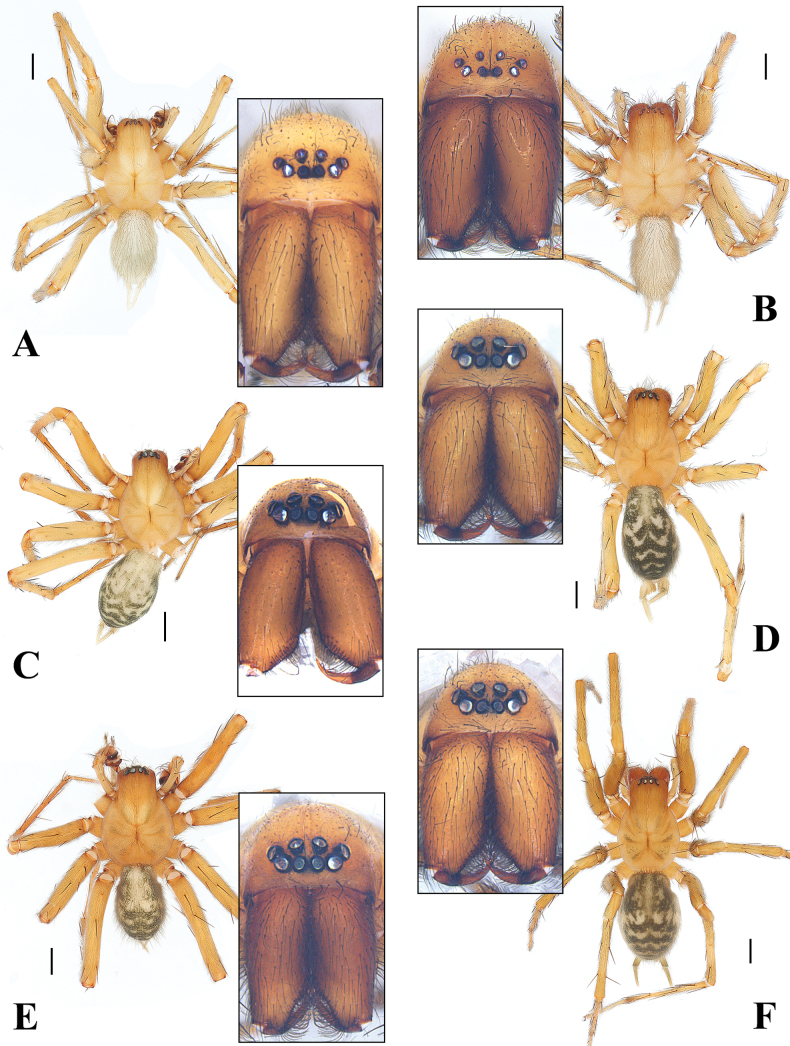
Habitus of *Spiricoelotes* spp., dorsal view. **A, B.***S.metyr* sp. nov.; **C, D.***S.zhengi* sp. nov.; **E, F.***S.zonatus*. **A, C, E.** Males; **B, D, F.** Females. The subimages indicate the ocular area, frontal view. Scale bars: 1.00 mm.

**Figure 8.**
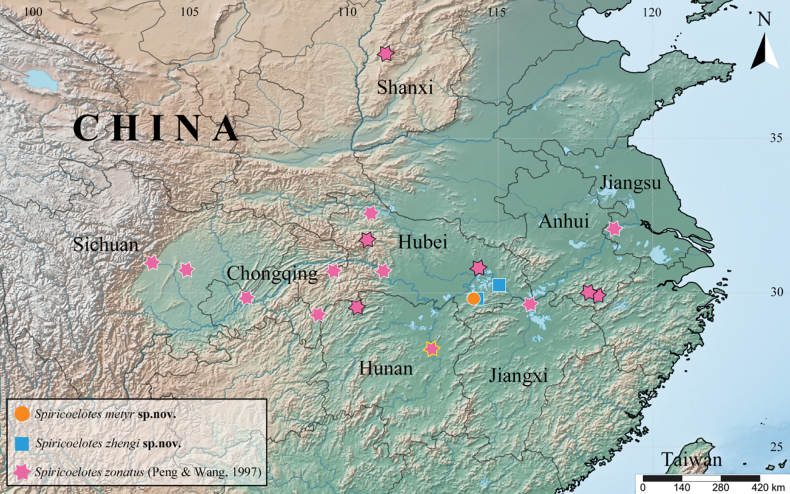
Distributions of *Spiricoelotes* spp. mentioned in the current paper of China. The hexagrams with white border indicate the localities of the examined materials, with black border indicate the localities of other unexamined materials, and with yellow border indicate the type locality.

The males of *Spiricoelotesmetyr* sp. nov. can be easily distinguished from all other congeners in 1) having a leaf-shaped conductor in dorsal view, with the distal tip of the conductor thin, long, and pointed downward (Fig. 1A, B), versus being not leaf-shaped, sometimes coiled, but always pointed upward in other congeners (Fig. 3B, E; fig. 25 in Shimojana 1989; figs 1B, 3B, 5B, 7B, 9B in Chen et al. 2016); 2) the patellar apophysis extremely strong and long (Fig. 1B, C), versus being relatively thin and short in other congeners (Fig. 3C, F; fig. 25 in Shimojana 1989; figs 1C, 3C, 5C, 7C, 9C in Chen et al. 2016); 3) the cymbial furrow extremely long, approximately 4/5 the length of the cymbium (Fig. 1C), versus being subequal to or less than 1/2 the length of the cymbium in other congeners (Fig. 3C, F; fig. 25 in Shimojana 1989; figs 1C, 3C, 5C, 7C, 9C in Chen et al. 2016). The females of the new species resemble those of *S.xiongxinensis* in 1) having laterally situated copulatory openings that are far apart, and extremely short copulatory ducts (Fig. 2A, B; fig. 11A, B in Chen et al. 2016); 2) shallow and wide hoods (Fig. 2A; fig. 11A in Chen et al. 2016); 3) spermathecae that are not coiled, with the length of the spermatheca being subequal to the length of the epigynal plate (Fig. 2B; fig. 11B in Chen et al. 2016). In other congeners, the copulatory openings are situated relatively medially and are close to each other (Figs 5A, B, 6A, B; figs 28, 29 in Shimojana 1989; figs 2A, B, 6A, B, 8A, B, 10A, B in Chen et al. 2016), or laterally situated but with long copulatory ducts in *S.chufengensis* (fig. 4A, B in Chen et al. 2016); the hoods deep and thin (figs 5A, 6A; fig. 28 in Shimojana 1989; figs 2A, 4A, 6A, 8A, 10A in Chen et al. 2016); the spermathecae coiled (Figs 5B, 6B; fig. 29 in Shimojana 1989), or not coiled but less than half the length of the epigynal plate (figs 2B, 4B, 6B, 8B, 10B in Chen et al. 2016). But the new species can be differentiated from *S.xiongxinensis* in having relatively thin, regularly shaped spermathecae (Fig. 2B), versus thick and irregularly shaped in *S.xiongxinensis* (fig. 11B in Chen et al. 2016).

##### Description.

**Male holotype** (Fig. 7A). Carapace yellowish, cervical and radial grooves indistinct. Chelicerae with 3 promarginal teeth and 5 retromarginal teeth, condyle weak. Sternum longer than wide. Abdomen nearly white, without patterns, covered by blueish gray hairs. Legs yellowish. Total length 6.70. Carapace 3.86 long, 2.69 wide. Abdomen 2.78 long, 1.82 wide. Eye diameters and interdistances: AME 0.16, ALE 0.13, PME 0.15, PLE 0.12; AME–AME 0.07, AME–ALE 0.08, PME–PME 0.14, PME–PLE 0.10. Measurements of legs: I 14.81 (4.06, 1.27, 3.50, 3.68, 2.30), II 13.04 (3.68, 1.14, 3.26, 2.73, 2.23), III 13.00 (3.49, 0.68, 2.92, 3.74, 2.17), IV 16.25 (4.32, 0.82, 4.38, 4.16, 2.57).

***Palp*** (Fig. 1). Patellar apophysis long, as twice long as the length of patella. Retrolateral tibial apophysis subequal to the length of tibia. Lateral tibial apophysis short, subequal to 1/5 the length of retrolateral tibial apophysis. Cymbial furrow long, subequal to 4/5 the length of cymbium. Conductor leaf-shaped from ventral view; ventral margin short and strongly sclerotized; dorsal margin wide, distal tip thin and long, pointed downward, with developed membranous ridge; apophysis of dorsal margin broad and ridge-shaped. Tegular ridge slice-shaped and situated retroalterally, matched with the ridge-shaped dorsal apophysis of conductor. Embolus arising in a 4:00–4:30 o’clock-position, extremely long. Median apophysis reduced.

**Female paratype** (Fig. 7B). Same in colour, abdominal patterns, and chelicera teeth as male. Total length 7.89. Carapace 3.99 long, 2.56 wide. Abdomen 3.67 long, 2.61 wide. Eye diameters and interdistances: AME 0.15, ALE 0.14, PME 0.16, PLE 0.15; AME–AME 0.09, AME–ALE 0.11, PME–PME 0.11, PME–PLE 0.10. Measurements of legs: I 13. 56 (3.53, 1.16, 3.04, 3.56, 2.27), II 12. 52 (3.37, 1.31, 3.12, 2.92, 1.80), III 11. 42 (3. 17, 0.48, 2.90, 3.30, 1.57), IV 16. 13 (4.14, 0.97, 3.82, 4.81, 2.39).

***Epigyne*** (Fig. 2). Epigynal plate wider than long. Copulatory openings situated laterally and separated from each other. Hoods located anteriorly. Copulatory ducts extremely short. Spermathecal heads small, situated near the beginning of spermathecal stalks; spermathecae curved-finger-shaped, with smooth surface and with extremely coiled duct inside. Fertilization ducts short, posteriorly situated.

##### Variation.

The deep hoods appear in only one female individual from Daquan Cave, and we consider it to be a mutation.

##### Distribution.

Only known from the type localities (Fig. 8).

#### ﻿ Spiricoelotes zhengi sp. nov.

Taxon classificationAnimaliaAraneaeAgelenidae

37DCE5E0-0DB8-590F-BBEB-81E88C81F0CD

https://zoobank.org/4EB13835-F587-4A6F-BAD7-E5E4675BA007

Figs 3A–C , 4C, D , 5 , 7C, D , 8

##### Type materials.

***Holotype*** • ♂ (CBEE, LJ202385), **China: *Hubei Province***: Xianning City, Chongyang County, entrance of Daquan Cave, 29.5534°N, 114.2662°E, elevation: 124 m, 31.X.2023, Jian Chang, Mian Wei, Guoyuan Zhang and Haosiyi Zhu leg. ***Paratypes***: • 2♂♂4♀♀(CBEE, LJ202386–LJ202391), same data as holotype; • 1♀(CBEE, LJ202392), **China: *Hubei Province***: Huangshi City, Daye County, entrance of East Cave, 30.1800°N, 115.1043°E, elevation: 174 m, 26.XI.2023, Jian Chang, Hailun Chen, Jie Liu, Zhuoning Liu and Mian Wei leg.; • 1♀(CBEE, LJ202393), **China: *Hubei Province***: Huangshi City, Daye County, West Cave, 30.1789°N, 115.1027°E, elevation: 165 m, 26.XI.2023, Jian Chang, Hailun Chen, Jie Liu, Zhuoning Liu and Mian Wei leg.; • 3♂♂3♀♀(CBEE, LJ202394–LJ202399), **China: *Hubei Province***: Xianning City, Xianan District, entrance of a nameless cave, 29.7715°N, 114.3122°E, elevation: 89 m, 10.XII.2023, Jian Chang, Guolong Huang and Mian Wei leg.

##### Etymology.

The specific name is dedicated to Yuandong Zheng, in appreciation of his specimen donations to our research group; this name is treated as a noun (name) in the genitive case.

##### Diagnosis.

*Spiricoeloteszhengi* sp. nov. resembles *S.zonatus* and *S.urumensis*. The males resemble those of the latter in 1) having a spiculate and spiral distal part of the dorsal margin of the conductor (Figs 3B, E, 4D, F; figs 25, 26 in Shimojana 1989); 2) the cymbial furrow being deep and relatively long, measuring no less than 1/3 or subequal to 1/2 the length of the cymbium (Fig. 3C, F; fig. 26 in Shimojana 1989). In other congeners, the conductor is thick and short (fig. 7 in Chen et al. 2016), or long and sometimes coiled but not spiral (figs 1B, 3B, 5B, 9B in Chen et al. 2016); the cymbial furrow is less than 1/3 the length of the cymbium (figs 1C, 3C, 5C, 7C, 9C in Chen et al. 2016), or extremely long, subequal to 4/5 the length of the cymbium (Fig. 1C). However, *S.zhengi* sp. nov. can be distinguished from the latter by 1) having a relatively short cymbial furrow, less than 1/2 the length of the cymbium (Fig. 3C), versus being subequal to 1/2 the length of the cymbium in *S.zonatus* (Fig. 3F); 2) the patellar apophysis is shorter than the patella (Fig. 3C), versus being subequal to the length of the patella in *S.zonatus* (Fig. 3F), or obviously longer than the patella in *S.urumensis* (fig. 26 in Shimojana 1989); 3) the dorsal margin of the conductor is broad, with the spiral part of the dorsal margin of the conductor relatively short (Fig. 3A–C), versus being relatively thin in *S.zonatus* (Fig. 3D–F), or having an extremely long spiral part in *S.urumensis* (figs 25, 26 in Shimojana 1989); 4) the embolus is relatively short (Figs 3A, B, 4C, D), versus being long in *S.zonatus* (Figs 3D, E, 4E, F). The females of the new species resemble those of *S.zonatus* in 1) lacking an atrium and having large, round copulatory openings (Figs 5A, 6A); 2) spermathecal stalk being long and coiled (Figs 5B, 6B). In other congeners, the atria are weak and separated, and the copulatory openings are small or large, but irregularly shaped (fig. 2A; fig. 28 in Shimojana 1989; figs 2A, B, 4A, B, 6A, B, 8A, B, 10A, B, 11A, B in Chen et al. 2016); the spermathecal stalks are short and not coiled (fig. 29 in Shimojana 1989; figs 2B, 4B, 6B, 8B, 10B, 11B in Chen et al. 2016). However, *S.zhengi* sp. nov. can be distinguished from *S.zonatus* in having 1) relatively short copulatory ducts, which are almost covered by the coiled spermathecal stalk (Fig. 5B), versus are relatively long in *S.zonatus* (Fig. 6B); 2) thick spermathecal stalks and the posterior part of the spermathecal stalk with a right-angled turn (Fig. 5B), versus spermathecal stalks relatively thin and the posterior part of the spermathecal stalk has a U-shaped turn in *S.zonatus* (Fig. 6B); 3) the spermathecal bases are nearly capsule-shaped (Fig. 5B), versus are mildly curved and kidney-shaped in *S.zonatus* (Fig. 6B).

##### Description.

**Male holotype** (Fig. 7C). Carapace yellowish, cervical and radial grooves indistinct, with weak patterns. Chelicerae with 3 promarginal teeth and 5 retromarginal teeth, condyle weak. Sternum longer than wide. Abdomen dark, with 6 yellowish chevron-shaped patterns, covered by blueish gray hairs. Legs yellowish. Total length 6.82. Carapace 3.43 long, 2.50 wide. Abdomen 3.20 long, 2.16 wide. Eye diameters and interdistances: AME 0.11, ALE 0.16, PME 0.16, PLE 0.16; AME–AME 0.08, AME–ALE 0.03, PME–PME 0.10, PME–PLE 0.09. Measurements of legs: I 13. 72 (3.83, 1.07, 3.66, 3.35, 1.81), II 13. 31 (3.74, 0.93, 3.70, 2.80, 2.14), III 12. 30 (3.27, 0.58, 2.69, 3.37, 1.64), IV 15. 25 (3.79, 0.92, 3.65, 4.67, 2.22). Chelicera with 3 promarginal and 5 retromarginal teeth.

***Palp*** (Fig. 3A–C). Patellar apophysis approximately 2/3 length of patella, hook-shaped, and with distal tip strongly curved backward. Retrolateral tibia apophysis with semicircular tip. Lateral tibial apophysis short, approximately 1/3 length of retrolateral tibia apophysis. Cymbial furrow long, approximately 2/5 the length of cymbium. Conductor pointed upward, ventral margin short and sclerotized; dorsal margin consisted with a smooth basal part and a spiral distal part, and with a membranous ridge; apophysis of dorsal margin of conductor ridge-shaped. Tegulum with a large slice-shaped ridge situated retrolaterally, matched with the ridge-shaped dorsal apophysis of conductor. Embolus arising in a 5:00–5:30 o’clock-position. Median apohysis absent.

**Female paratype** (Fig. 7D). Same in abdominal patterns and chelicera teeth as male but in darker colour. Total length 7.48. Carapace 3.62 long, 2.68 wide. Abdomen 3.78 long, 2.30 wide. Eye diameters and interdistances: AME 0.15, ALE 0.13, PME 0.17, PLE 0.15; AME–AME 0.07, AME–ALE 0.12, PME–PME 0.10, PME–PLE 0.10. Measurements of legs: I 13. 59 (3.17, 0.98, 3.83, 3.41, 2.20), II 12. 79 (3.45, 1.31, 2.89, 3.16, 1.89), III 11. 37 (3.16, 0.83, 3.34, 2.36, 1.68), IV 15. 04 (4.03, 1.10, 3.08, 4.70, 2.13).

***Epigyne*** (Fig. 5). Epigynal plate wider than long. Atrium absent. Copulatory openings large and distinct, located antero-medially. Hoods distinct, located laterally, above copulatory openings. Copulatory ducts short and membranous, nearly hidden by spermathecal stalks. Spermathecal heads small, situated near the beginning of spermathecal stalks; spermathecal stalks long and coiled twice, posterior part with a U-shaped turn; spermathecal bases capsule-shaped. Fertilization ducts short, posteriorly situated.

##### Variation.

The median apophysis present in some individuals as a small, sclerotized patch.

##### Distribution.

Only known from the type localities (Fig. 8).

#### ﻿ Spiricoelotes zonatus

Taxon classificationAnimaliaAraneaeAgelenidae

(Peng & Wang, 1997)

B40A1FCD-32EA-563A-8246-7B71559DAEDC

Figs 3D–F , 4E, F , 6 , 7E, F , 8

Coelotes zonatus Peng & Wang, 1997: 331, figs 32–36; Song et al. 1999: 388, figs 226O, P, 227P, 229B.
Coelotes laoyingensis Chen & Zhao, 1997: 89, figs 5, 6; Song et al. 1999: 376, fig. 220N, O.
Spiricoelotes zonatus : Wang 2002:131, figs 360–374; Wang 2003: 565, figs 80A–E, 97I; Okumura 2008: 1, figs 1–4; Okumura et al. 2009: 200, figs 406–409; Zhu and Zhang 2011: 330, fig. 239A–E; Yin et al. 2012: 1028, fig. 533a–e; Zhu et al. 2017: 531, fig. 350A–E.

##### Type materials

**(not examined). *Holotype*** • ♀ (HNNU), **China: *Hunan Province***: Changsha City, Mt Yuelu, 17.I.1983, Jiafu Wang leg. ***Paratypes***: 2 ♂♂5 ♀♀ (HNNU), same data as the holotype.

##### Materials examined.

• 3♀♀ (CBEE, LJEX01–LJEX03, type materials of *Coeloteslaoyingensis*), **China: *Hubei Province***: Mt Wudang, Laoying, 10.V.1982; • 5♂♂7♀♀ (CBEE, LJSC01–LJSC12), **China: *Sichuan Province***: Chengdu City, Shuangliu District, Jiang’an Campus of Sichuan University, 30.5518°N, 103.9963°E, elevation: 477 m, XI.2018, Mian Wei leg.; • 1♂5♀♀ (CBEE, LJSC13–LJSC18), **China: *Sichuan Province***: Dujiangyan City, Mt Qingchengshan, 30.9319°N, 103.4838°E, elevation: 1323 m, V.2019, Mian Wei leg.; • 12♂♂9♀ (CBEE, LJJS01–LJJS21), **China: *Jiangsu Province***: Nanjing City, Xuanwu District, Mt Zijinshan, 32.0704°N, 118.8533°E, elevation: 225 m, 10.I.2020, Mian Wei leg.; • 3♂♂2♀♀ (CBEE, LJHB01–LJHB05), **China: *Hubei Province***: Yichang City, Dianjun District, Mojishan Forest Park, 30.6822°N, 111.2796°E, elevation: 71 m, 16.II.2021, Mian Wei leg.; • 2♂♂2♀♀ (CBEE, LJCQ01–LJCQ04), **China: *Chongqing Municipality***: Yubei District, Jiuquhe Wetland Park, 29.6533°N, 106.5072°E, elevation: 247 m, 16.XI.2022, Hailun Chen leg.; • 2♂♂1♀ (CBEE, LJHB06–08), **China: *Hubei Province***: Enshi Tujia and Miao Autonomous Region, Jianshi County, entrance of Pufeng Cave, 30.5622°N, 109.6953°E, elevation: 657 m, 10.X.2023, Jiang Chang, Guolong Huang and Mian Wei leg.; • 10♂♂17♀♀ (CBEE, LJHB09–LJHB35), **China: *Hubei Province***: Enshi Tujia and Miao Autonomous Region, Laifeng County, entrance of Feng Cave, 29.1684°N, 109.1937°E, elevation: 414 m, 12.X.2023, Jiang Chang, Guolong Huang and Mian Wei leg.; • 1♀ (CBEE, LJHB36), **China: *Jiangxi Province***: Jiujiang City, Mt. Lushan, 29.5495°N, 115.9920°E, elevation: 1145 m, 1.XII.2023, local collector leg.

##### Diagnosis.

*Spiricoeloteszonatus* resembles *S.urumensis* and *S.zhengi* sp. nov. See diagnosis of *S.zhengi* sp. nov. above.

##### Description.

See Zhu et al. (2017: fig. 350A–E) for detailed description.

##### Distribution.

China (Anhui, Chongqing, Hubei, Hunan, Jiangsu, Jiangxi, Shanxi, Sichuan) (Fig. 8), introduced to Japan (Nagasaki) (Okumura, 2008).

## Supplementary Material

XML Treatment for Spiricoelotes

XML Treatment for Spiricoelotes metyr

XML Treatment for Spiricoelotes zhengi

XML Treatment for Spiricoelotes zonatus

## ﻿Additional information

### Use of AI

No use of AI was reported.

### Author ORCIDs

Hailun Chen https://orcid.org/0009-0009-0879-6887

Jie Liu https://orcid.org/0000-0001-7744-9744

Mian Wei https://orcid.org/0000-0001-7348-8885

### Data availability

All of the data that support the findings of this study are available in the main text.
